# ZNF692 promotes osteosarcoma cell proliferation, migration, and invasion through TNK2-mediated activation of the MEK/ERK pathway

**DOI:** 10.1186/s13062-024-00472-3

**Published:** 2024-04-22

**Authors:** Di Zheng, Zhun Wei, Chong Zhang, Wenda Liu, Changtian Gong, Fei Wu, Weichun Guo

**Affiliations:** Department of Orthopedics, Renmin Hospital of Wuhan University, 430060 Wuhan, China

**Keywords:** ZNF692, Osteosarcoma, MEK/ERK pathway, TNK2, Proliferation

## Abstract

**Background:**

Osteosarcoma is a diverse and aggressive bone tumor. Driver genes regulating osteosarcoma initiation and progression remains incompletely defined. Zinc finger protein 692 (ZNF692), a kind of Krüppel C2H2 zinc finger transcription factor, exhibited abnormal expression in different types of malignancies and showed a correlation with the clinical prognosis of patients as well as the aggressive characteristics of cancer cells. Nevertheless, its specific role in osteosarcoma is still not well understood.

**Methods:**

We investigated the dysregulation and clinical significance of ZNF692 in osteosarcoma through bioinformatic method and experimental validation. A range of in *vitro* assays, including CCK-8, colony formation, EdU incorporation, wound healing, and transwell invasion tests, were conducted to assess the impact of ZNF692 on cell proliferation, migration, and invasion in osteosarcoma. A xenograft mouse model was established to evaluate the effect of ZNF692 on tumor growth in vivo. Western blot assay was used to measure the protein levels of MEK1/2, P-MEK1/2, ERK1/2, and P-ERK1/2 in cells that had been genetically modified to either reduce or increase the expression of ZNF692. The relationship between ZNF692 and tyrosine kinase non-receptor 2 (TNK2) were validated by qRT-PCR, chromatin immunoprecipitation and luciferase reporter assays.

**Results:**

Expression of ZNF692 was increased in both human osteosarcoma tissues and cell lines. Furthermore, the expression of ZNF692 served as an independent predictive biomarker in osteosarcoma. The results of the survival analysis indicated that increased expression of ZNF692 was associated with worse outcome. Downregulation of ZNF692 inhibits the proliferation, migration, and invasion of osteosarcoma cells, whereas upregulation of ZNF692 has the opposite impact. Western blot assay indicates that reducing ZNF692 decreases phosphorylation of MEK1/2 and ERK1/2, whereas increasing ZNF692 expression enhances their phosphorylation. U0126, a potent inhibitor specifically targeting the MEK/ERK signaling pathway, partially counteracts the impact of ZNF692 overexpression on the proliferation, migration, and invasion of osteosarcoma cells. In addition, ZNF692 specifically interacts with the promoter region of TNK2 and stimulates the transcription of TNK2 in osteosarcoma cells. Forcing the expression of TNK2 weakens the inhibitory impact of ZNF692 knockdown on P-MEK1/2 and P-ERK1/2. Similarly, partly inhibiting TNK2 counteracts the enhancing impact of ZNF692 overexpression on the phosphorylation of MEK1/2 and ERK1/2. Functional tests demonstrate that the suppressive effects of ZNF692 knockdown on cell proliferation, migration, and invasion are greatly reduced when TNK2 is overexpressed. In contrast, the reduction of TNK2 hinders the ability of ZNF692 overexpression to enhance cell proliferation, migration, and invasion.

**Conclusion:**

ZNF692 promotes the proliferation, migration, and invasion of osteosarcoma cells via the TNK2-dependent stimulation of the MEK/ERK signaling pathway. The ZNF692-TNK2 axis might potentially function as a possible predictive biomarker and a promising target for novel therapeutics in osteosarcoma.

**Supplementary Information:**

The online version contains supplementary material available at 10.1186/s13062-024-00472-3.

## Introduction

Osteosarcoma, which mostly affects children, teenagers, and young adults, is the most prevalent malignant bone tumor. The incidence is approximately three to five cases per million individuals annually [1, 2]. Osteosarcoma primarily affects the long bones that support mechanical stress, with the distal femur, proximal tibia, and humerus being the three most often affected locations [3]. Individuals diagnosed with osteosarcoma often have pronounced pain and inflammation in the afflicted bones, and in some instances, it may result in pathological fractures. The current standard treatment for osteosarcoma patients involves limb-sparing surgery, neoadjuvant chemotherapy, and adjuvant chemotherapy. This treatment approach has significantly improved the five-year overall survival rate for patients with localized osteosarcoma, increasing it from less than 20% to over 60% [4, 5]. Regrettably, about 20–30% are metastatic or recurrent cases due to its highly malignancy, and it is worth noting that approximately 15–20% patients have metastases at diagnosis, with the lung parenchyma being the most frequent location [6, 7]. Despite intensive research, the prognosis for patients with metastatic osteosarcoma is still poor [8]. In addition, the adverse effects of chemotherapy drugs and the acquired-resistance also lead to the failure of treatment [9, 10]. Given these barriers to conventional treatment regimens, there is an urgent need to investigate the regulatory mechanism underlying tumor cell proliferation and metastasis, which might lead to the identification of novel molecular targets and approaches for diagnosis, prognosis prediction, and treatment.

Zinc finger (ZNF) proteins are a group of regulatory proteins that include zinc ion-binding finger-like domains. These proteins play various roles in human illnesses, such as cancer, by either activating or suppressing the expression of their target genes [11–13]. The gene *ZNF692*, which encodes the zinc finger protein 692, is also referred to as *AREBP* and *Zfp692*. It is situated on the chromosomal region 1q44. Previous studies have shown abnormal ZNF692 expression in many types of malignancies, and it has been linked to patients’ clinical prognosis and the aggressive characteristics of cancer cells [14–16]. For instance, the excessive expression of *ZNF692* was shown to be an independent risk factor for worse overall survival in lung adenocarcinoma (LUAD), and in vitro and in vivo experiments indicated that suppressing ZNF692 hindered cell proliferation, migration, and invasion of LUAD cells [14]. *ZNF692* overexpression in cervical cancer is linked to unfavorable clinicopathological features. Functional investigations have shown that ZNF692 regulates the proliferation and invasion of cervical cancer cells by suppressing the transcription of p27^kip1^ [15]. *ZNF692* expression was shown to be significantly associated with tumor stage and metastasis in colon adenocarcinoma (COAD). Moreover, the overexpression of ZNF692 was observed to enhance cell proliferation and metastasis via activating the PI3K/AKT pathway [16]. These studies indicate that ZNF692 may serve as a prognostic biomarker and operate as an oncoprotein in malignant tumors. However, its involvement in osteosarcoma has not been studied yet.

There are four distinct MAPK pathways, namely the classical ERK, c-Jun N-terminal kinase (JNK), p38 signaling families, and the unclassical Big MAP kinase-1 (BMK-1). These pathways play a crucial role in regulating various cellular processes such as proliferation, differentiation, apoptosis, and stress responses [17, 18]. Generically, the MAPK/ERK signal is activated by a variety of internal and external stimuli, such as metabolic stress and DNA damage pathways, as well as signaling from external growth factors and cell-cell communications [19, 20]. The internal and external inputs trigger a cascade of kinases, ranging from MAPK kinase-kinase (A-RAF, B-RAF and C-RAF) to MAPK kinase (MEK1 and MEK2), and ultimately leading to the activation of the final effector molecules ERK1 and ERK2 [21]. Upon activation, ERK1 and ERK2 migrate to the nucleus and catalyze the phosphorylation of certain crucial substrates involved in cellular processes such as proliferation, cell cycle regulation, differentiation, and cell death [22, 23].

Here, our investigation reveals a consistent up-regulation of ZNF692 in osteosarcoma, and establishes a noteworthy correlation between elevated *ZNF692* expression and worse clinical prognosis in osteosarcoma patients. Functional experiments indicates that ZNF692 has a tumor-promoting function in osteosarcoma. Additionally, overexpression of ZNF692 stimulates the proliferation, migration, and invasion of osteosarcoma cells. ZNF692 stimulates tyrosine kinase non-receptor 2 (TNK2) via transcriptional mechanisms, resulting in the subsequent activation of the MEK/ERK signaling cascade. In summary, our research reveals that *ZNF692* may serve as a novel prognostic indicator and oncogene in osteosarcoma.

## Materials and methods

### Sample collection

Osteosarcoma tissues and adjacent normal tissues were obtained from Renmin hospital of Wuhan university between June 2021 to June 2023. Detailed basic information of clinical samples is shown in Table 1. All the tissue samples were harvested after surgical resection and stored in liquid nitrogen for further experiments. The experiment with patient tissue specimens was authorized by the Ethics Committee of Renmin Hospital of Wuhan University.

**Table 1:** Correlation analyses of ZNF692 protein expression in relation to clinicopathologic variables of patients with osteosarcoma

Characteristics	Total	ZNF692 expression		*P* value
		Low	High	
Gender				*P* = 0.2557
Male Female	15 15	4 7	11 8	
Age				*P* = 0.666
≤18 years >18 years	23 7	12 3	11 4	
Recurrence				*P =* 0.0074**
Yes No	19 11	3 7	16 4	
Metastasis				*P =* 0.0112*
Yes No	21 9	4 6	17 3	
TNM phase				*P =* 0.0441*
I/II III/IV	6 24	1 15	5 9	

### Bioinformatic analysis

Osteosarcoma cohort, comprising the RNA sequencing (RNA-seq) data and corresponding clinical information, was downloaded from The Cancer Genome Atlas (TCGA) database (https://portal.gdc.cancer.gov/) and was utilized to evaluate the prognostic prediction performance of ZNF692. GSE126209 and GSE16091 datasets were download from the Gene Expression Omnibus (GEO) database (https://www.ncbi.nlm.nih.gov/geo/). GSE126209 dataset contained 12 osteosarcoma tissues and 11 adjacent normal tissues and was used to compare the expression level of ZNF692 in osteosarcoma tissues and adjacent normal tissues. GSE16091 dataset and the GEPIA database (http://gepia.cancer-pku.cn/detail.php) were used to analyze the correlation between ZNF692 and TNK2 in osteosarcoma samples or other types of tumor samples, respectively. The TMIER database (https://cistrome.shinyapps.io/timer/) was used to analyzed the expression profiles of *ZNF692* in various malignant tumors.

### Cell culture

The human osteosarcoma cell lines 143B, MG63 and HOS were purchased from the Cell Bank of Wuhan University (Wuhan, China). The U2OS and Saos-2 were purchased from the Cell Bank of Type Culture Collection (CBTCC, Chinese Academy of Sciences, Shanghai, China). Human osteoblasts (hFOB1.19) were purchased from ProCell Technology (Wuhan, China). 143B and HOS cells were cultured in α-MEM. U2OS and Saos-2 cells were cultured in McCoy’s 5 A medium. MG63 and hFOB1.19 cells were cultured in RPMI 1640 medium. All culture media were supplemented with 10% fetal bovine serum (FBS) and 1% antibiotics (100 U/ml penicillin and 100 mg/ml streptomycin). Osteosarcoma cells were maintained in a humidified chamber with 5% CO_2_ at 37℃, while the incubation temperature of hFOB1.19 was 34 °C.

### Transfection

For lentivirus infection, shRNA specifically targeting *ZNF692* (shZNF692), negative control shRNA (shNC), *ZNF692* overexpression lentivirus (LV-ZNF692) and empty vector (LV-Control) were obtained from OBiO Technology (Shanghai, China), and were then utilized to infect 143B and U2OS osteosarcoma cells according to the manufacturer’s instructions. Osteosarcoma cells of logarithmic growth stage were taken 24 h before infection, and the cells were inoculated into a six-well plate at a rate of 1 × 10^5^ cells/well, and cultured in a cell incubator at 37 °C and 5% CO_2_. Then, according to the MOI value of the virus in the instructions, the required volume of the virus was calculated for infection. After the operation was completed, the six-well plate was placed at 37 °C and cultured in a cell incubator at 5% CO_2_. After continuing the culture for 12 h, the medium was discarded, cleaned with PBS, and replaced with a new complete medium for further culture. The osteosarcoma cells were screened by adding puromycin (5 µg/ mL) 2 days after infection and were confirmed by qRT-PCR and western blotting. For siRNA or plasmids transfection, siRNA targeting *TNK2* (siTNK2) and negative control siRNA (siNeg), *TNK2* overexpression vector (pcDNA3.1-TNK2-Flag) and empty vector were purchased from Sangon Biotech (Shanghai, China). siRNAs or plasmids were transfected into cells using Lipofectamine RNAiMAX or Lipofectamine 2000 according to the manufacturer’s protocol.

### CCK-8 and colony formation assays

Cell proliferation was detected by the CCK-8 and colony formation assays. Briefly, cells were harvested and resuspended in culture medium. For the CCK-8 assay, 1 × 10^4^ cells were seeded in 96-well plates and cultured for 24, 48, and 72 h. Then, 10 µL of CCK-8 (Cell Counting Kit-8) reagent was added into each well for the indicated times. The OD450 (optical density) value was measured with a plate reader. For the colony formation assay, 5 × 10^2^ cells were seeded in six-well plates and cultured for two weeks. The culture medium was replaced every three days. At the end of the experiments, the colonies were washed, fixed, dyed with 1% crystal violet, and photographed. The number of visible colonies were counted, and all experiments were repeated three times.

### 5-ethynyl-2’-deoxyuridine (Edu) assay

Briefly, osteosarcoma cells were incubated with Edu (50 µM) at 37 °C for 4 h. Then, cells were fixed with 4% formaldehyde for 15 min at room temperature. After permeabilization with 5% Triton X-100, cells were incubated with 100 µL of 1× Apollo®567 reaction cocktail for 30 min. Finally, cells were stained with 100 µL of Hoechst 33,342 for 30 min in the dark and photographed by light microscopy (Olympus, Tokyo, Japan).

### Wound healing assay

Osteosarcoma cells were seeded in six-well plates at a density of 1 × 10^6^ cells per well and cultured for 24 h to reach 90% confluence. Then, straight scratches were made on the plates using sterile pipette tips (200 µL), the cell debris was removed with PBS, and the serum-free medium was changed to inhibit cell proliferation. Images from the same position were photographed at 0 and 24 h by inverted microscopy (Olympus). Image J software was used to quantify the migration rate of osteosarcoma cells.

### Transwell invasion assay

Osteosarcoma cells were digested with trypsin and resuspended in serum-free medium at a density of 5 × 10^5^ cells/mL. 200 µL serum-free medium containing 1 × 10^5^ cells was added to the upper transwell chamber pre-coated with 60 µL Matrigel (Corning, Corning, NY). 500 µL complete culture medium was supplied in the lower chamber. After co-culture for 48 h, cells in the upper chamber were removed and cells in the bottom chamber were fixed in 4% paraformaldehyde, washed twice with PBS, stained with crystal violet solution, and finally photographed by light microscopy (Olympus). Image J software was used to quantify the invading cells.

### RNA extraction and qRT-PCR

Briefly, RNA was extracted using TRIzol reagent (Invitrogen, Carlsbad, CA) according to the manufacturer’s protocols. A total of 2 µg RNA was used to synthesize cDNA using the RevertAid First Strand cDNA Synthesis Kit (Thermo Fisher Scientific, Waltham, MA). Quantitative real time-polymerase chain reaction (qRT-PCR) was performed using SYBR Green Mix (Vazyme, Nanjing, China) to detect relative expression levels of target genes. mRNA expression of *GAPDH* was used as an internal control. The primer sequences were shown in Supplementary Table 1.

### Western blot analysis

Total proteins were extracted from tissues and cells using RIPA lysis buffer (Cell Signaling Technology, Danvers, MA) with protease and phosphatase inhibitors. Protein concentrations were quantified with a BCA kit (Beyotime Institute of Biotechnology, Beijing, China). 10 µg protein was separated by 10% or 12% sodium dodecyl sulfate-polyacrylamide gel electrophoresis (SDS-PAGE) before being transferred to polyvinylidene fluoride (PVDF) membranes. After blocking with milk at room temperature for 1 h, membranes were washed three times with TBST (Tris Buffered Saline with Tween-20) and incubated with specific antibodies overnight at 4 °C. The next day, membranes were incubated with secondary antibodies and the blots were then visualized with an ECL kit (Beyotime Institute of Biotechnology). Antibodies targeting ZNF692 were purchased from Abcam (1:500, ab204595, Cambridge, UK). Antibodies targeting GAPDH (1:500, GB13003), ERK1/2 (1:500, GB13003), P-ERK1/2 (1:500, GB11004) were purchased from Servicebio Technology (Wuhan, China). Antibodies targeting MEK1/2 (1:1000, #8727), P-MEK1/2 (1:1000, #2338) were purchased from Cell Signaling Technology (Danvers, MA). Antibodies targeting TNK2 were purchased from Santa Cruz Biotechnology (1:1000, sc-28,336).

### Chromatin immunoprecipitation assay

143B and U2OS cells stably overexpressing ZNF692 were collected. ChIP assays were conducted using the SimpleChIP® enzymatic ChIP kit (CST, #9002) according to the manufacturer’s protocol. The immunoprecipitated DNA were used as templates for ChIP-qPCR analysis. The primer sequences for ChIP were listed in Supplementary Table 2. The relative enrichment was calculated by percent input method.

### Luciferase reporter assay

The promoter region of *TNK2* genes and its mutant forms were inserted into the pGL3-Basic luciferase vector (Promega) to produce the luciferase reporter plasmids pGL3-TNK2-Luc. The coding sequences (CDS) region of *ZNF692* was cloned into the pcDNA3.1-flag vector to generate ZNF692-overexpression plasmid (pcDNA3.1-ZNF692-flag). 143B and U2OS cells were co-transfected with pcDNA3.1-ZNF692-flag or empty vector (pcDNA3.1-flag), pGL3-TNK2-Luc, and Renilla luciferase expression vector (pRL-TK). Cells were collected 48 h after transfection, the firefly and Renilla luciferase activities in the lysates were determined using a dual luciferase kit (Promega, cat#E1980). The relative luciferase activity was calculated by normalizing the firefly luciferase activity to that of the Renilla luciferase activity.

### Animal studies

Four-week-old female athymic mice, purchased from Beijing HFK Experiment Animal Center (Beijing, China), were housed in Animal Center of Wuhan University Renmin Hospital. All the mice were randomly divided into two groups and were then subcutaneously injected 143B cells stably knocking down *ZNF692* and control cells. Tumor growth was monitored every week by measuring the largest (a) and smallest (b) diameters of tumors. Tumor volume was calculated using the formula: tumor volume (mm^3^) = a×b^2^/2. All mice were sacrificed and the tumors were harvested and weighted. Tumors were then fixed with 4% paraformaldehyde or stored in liquid nitrogen for further experiments. All animal experiments were approved by the Animal Care and Use Committee of Wuhan University Renmin Hospital.

### Immunohistochemistry (IHC) staining

Immunohistochemistry (IHC) staining was conducted as previously described [24], and was utilized to evaluate the expression of Ki-67, ZNF692, TNK2, P-MEK, and P-ERK in xenograft tumors.

### Statistical analysis

R 4.1.0 software and GraphPad Prism 8.0 (La Jolla, CA) were used for statistical analyses. All data are presented as means ± standard deviations (SD). Student’s *t*-test and one-way ANOVA were performed to analyze differences between groups. A *P*-value < 0.05 was regarded as statistically significant.

## Results

### ZNF692 is upregulated in osteosarcoma and indicates poor prognosis in osteosarcoma patients

**Fig. 1:**
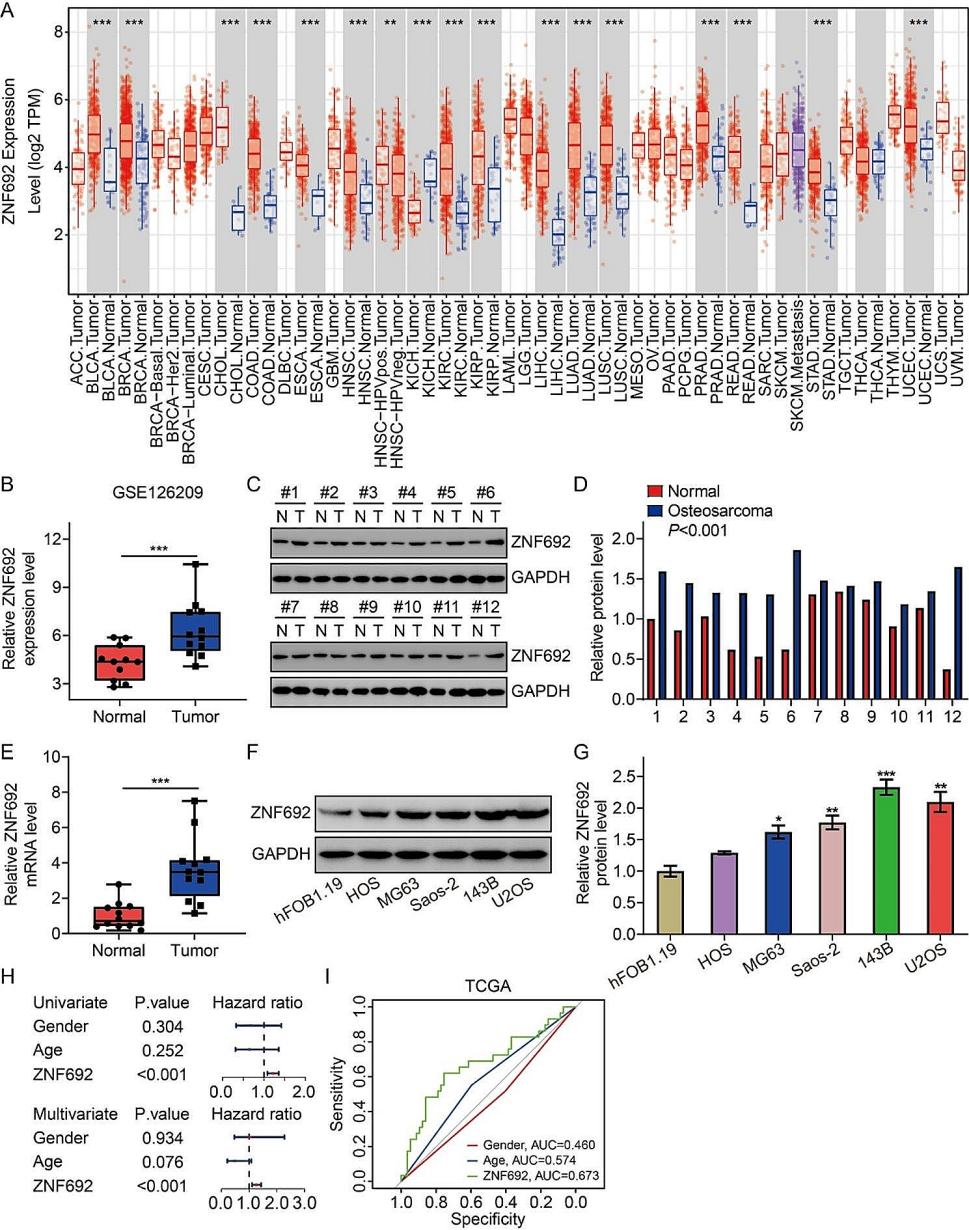
ZNF692 was upregulated in osteosarcoma and associated with worse overall survival. (**A**) The expression profiles of ZNF692 in various tumors based on TIMER database. (**B**) The transcript level of *ZNF692* in osteosarcoma tissues and adjacent normal tissues based on GSE126209 dataset. (**C-E**) ZNF692 expression was detected by western blot and qRT-PCR assay in osteosarcoma tissues and paired normal tissues. (**F-G**) ZNF692 protein level in osteosarcoma cell lines (HOS, MG63, U2OS, 143B, and Saos-2) and human osteoblast cells (FOB1.19) were detected by western blot assay, and quantitative analysis. (**H**) Univariate and multivariate Cox regression analyses were performed to evaluate the prognostic prediction ability of *ZNF692* in osteosarcoma. (**I**) ROC curve analysis was performed to assess the accuracy of *ZNF692* expression level, gender, and age in predicting patients’ prognosis in osteosarcoma. Student’s *t*-test and one-way ANOVA were performed to analyze differences between groups. All data are presented as means ± standard deviations (SD). *P < 0.05, **P < 0.01, ***P < 0.001

Initially, we examined the expression patterns of ZNF692 in different tumor types using the TIMER database (https://cistrome.shinyapps.io/timer/). Surprisingly, *ZNF692* expression was shown to be greatly increased in many malignancies such as BLCA, BRCA, CHOL, COAD, ESCA, HNCS, KIRC, KIRP, LIHC, LUAD, LUSC, PRAD, READ, STAD, and UCEC, when compared to their corresponding normal tissues. However, it was seen to be decreased in kidney chromophobe (KICH) (Fig. 1A). Regarding osteosarcoma, our analysis of the GSE126209 dataset from the GEO database revealed a significant upregulation of *ZNF692* expression in tumor tissues compared to normal tissues (Fig. 1B). We subsequently assessed the expression of *ZNF692* in osteosarcoma tissues using Western blot and qRT-PCR. Figure 1C-E demonstrate a significant increase in the protein and mRNA levels of ZNF692 in osteosarcoma tissues when compared to corresponding normal tissues. Furthermore, the levels of ZNF692 expression were significantly elevated in osteosarcoma cell lines (HOS, MG63, U2OS, 143B, and Saos-2) compared to human osteoblast cells (FOB1.19) (Fig. 1F-G). We further examine the prognostic predictive capability of *ZNF692* in osteosarcoma. Our study revealed that *ZNF692* was a potential risk factor in osteosarcoma in both univariate and multivariate Cox regression models, irrespective of clinical features such as gender and age (Fig. 1H). Furthermore, the area under the curve (AUC) values of the receiver operating characteristic (ROC) curves were 0.673, 0.574, and 0.460 for *ZNF692*, age, and gender, respectively. This indicates that *ZNF692* is quite accurate in predicting the prognosis of patients with osteosarcoma, as shown in Fig. 1I. Moreover, we have previously shown that elevated levels of ZNF692 were linked to worse prognosis in individuals with osteosarcoma [25]. Collectively, our data demonstrate that ZNF692 is elevated in osteosarcoma and is associated with a worse outcome in individuals with osteosarcoma.

### Silencing ZNF692 inhibits osteosarcoma cell proliferation, migration, and invasion

**Fig. 2:**
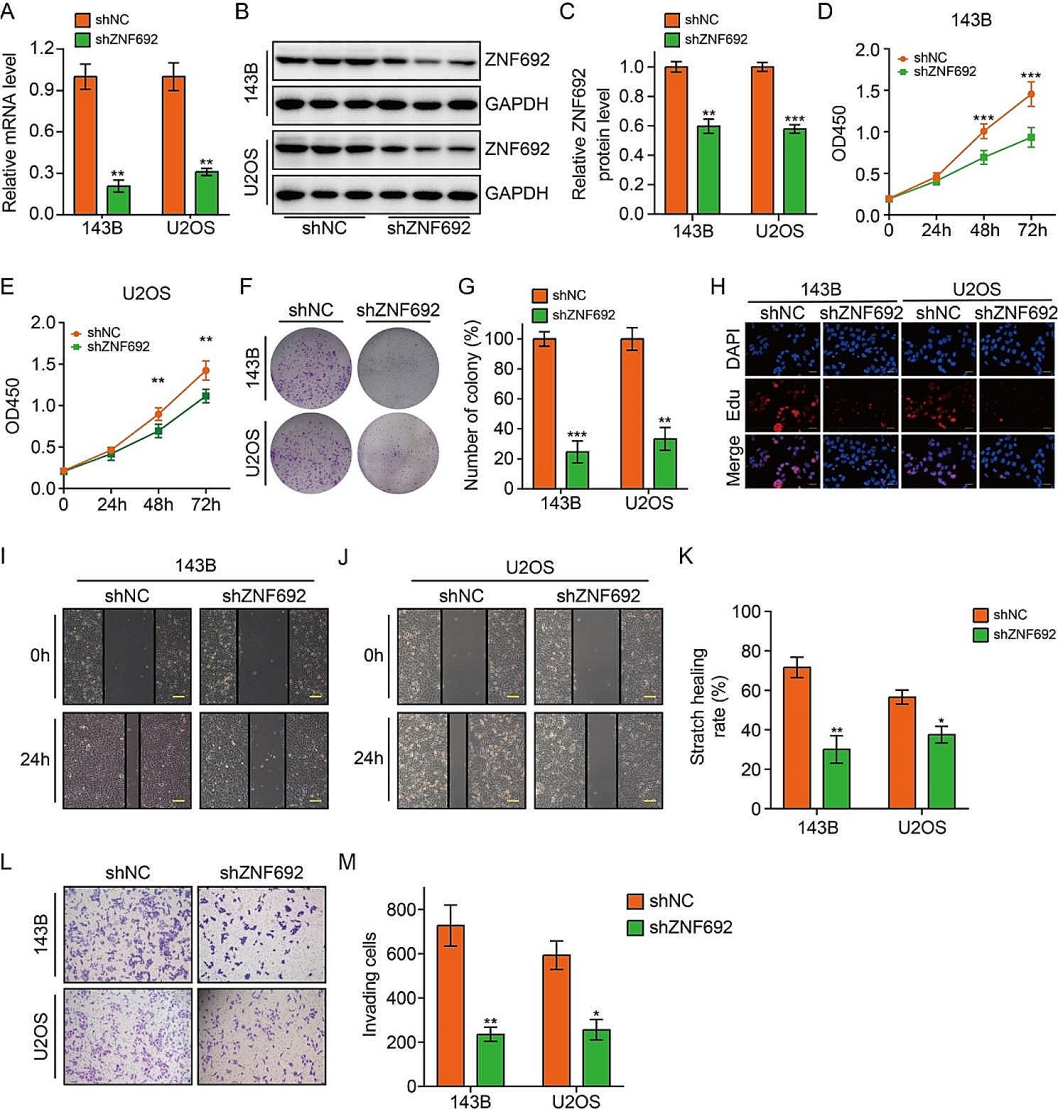
Silence of ZNF692 inhibits osteosarcoma cell proliferation, migration, and invasion. (**A-C**) Stably knocking down of ZNF692 was confirmed by qRT-PCR and western blot assays. (**D-E**) CCK-8 assay was performed to evaluate cell proliferation in cells stably knocking down of ZNF692 and control cells. (**F-G**) ZNF692 knock-down significantly impaired colony formation ability of osteosarcoma cells. (**H**) The proportion of Edu positive cell was decreased in osteosarcoma cells stably knocking down of ZNF692. Scale bar, 200 μm. (**I-K**) The migration ability of cells stably knocking down of ZNF692 and control cells was detected by wound healing assay (scale bar, 200 μm). (**L-M**) Representative images and quantitative analysis of cell invasion based on transwell assay. Student’s *t*-test and one-way ANOVA were performed to analyze differences between groups. All data are presented as means ± standard deviations (SD). *P < 0.05, **P < 0.01, ***P < 0.001

Cancer is characterized by the presence of sustained proliferative signals and the activation of invasion and metastasis, which are key features of the disease [26]. Thus, we investigated the potential impact of ZNF692 knockdown on cellular proliferation, migration, and invasion in osteosarcoma cells. Initially, we suppressed the expression of ZNF692 in 143B and U2OS cells using lentivirus infection. The reduction in ZNF692 was validated via qRT-PCR and Western blotting techniques (Fig. 2A-C). Subsequently, CCK-8, colony formation, and Edu incorporation assays were used to investigate the impact of ZNF692 silencing on the proliferation of osteosarcoma cells. Figure 2D-H demonstrate that cells transfected with shZNF692 exhibited considerably reduced OD450 values, colony numbers, and Edu-positive cells. This indicates that the knock-down of ZNF692 effectively suppressed cell growth. Stable silencing of ZNF692 in a wound-healing experiment resulted in a considerable decrease in the migration of 143B and U2OS cells (Fig. 2I-K). Similarly, downregulation of ZNF692 in a transwell invasion assay greatly hindered the invasion of osteosarcoma cells (Fig. 2L-M). Collectively, our findings indicate that the absence of ZNF692 inhibits the proliferation, migration, and invasion of osteosarcoma cells.

### Overexpression of ZNF692 promotes osteosarcoma cell proliferation, migration, and invasion

**Fig. 3:**
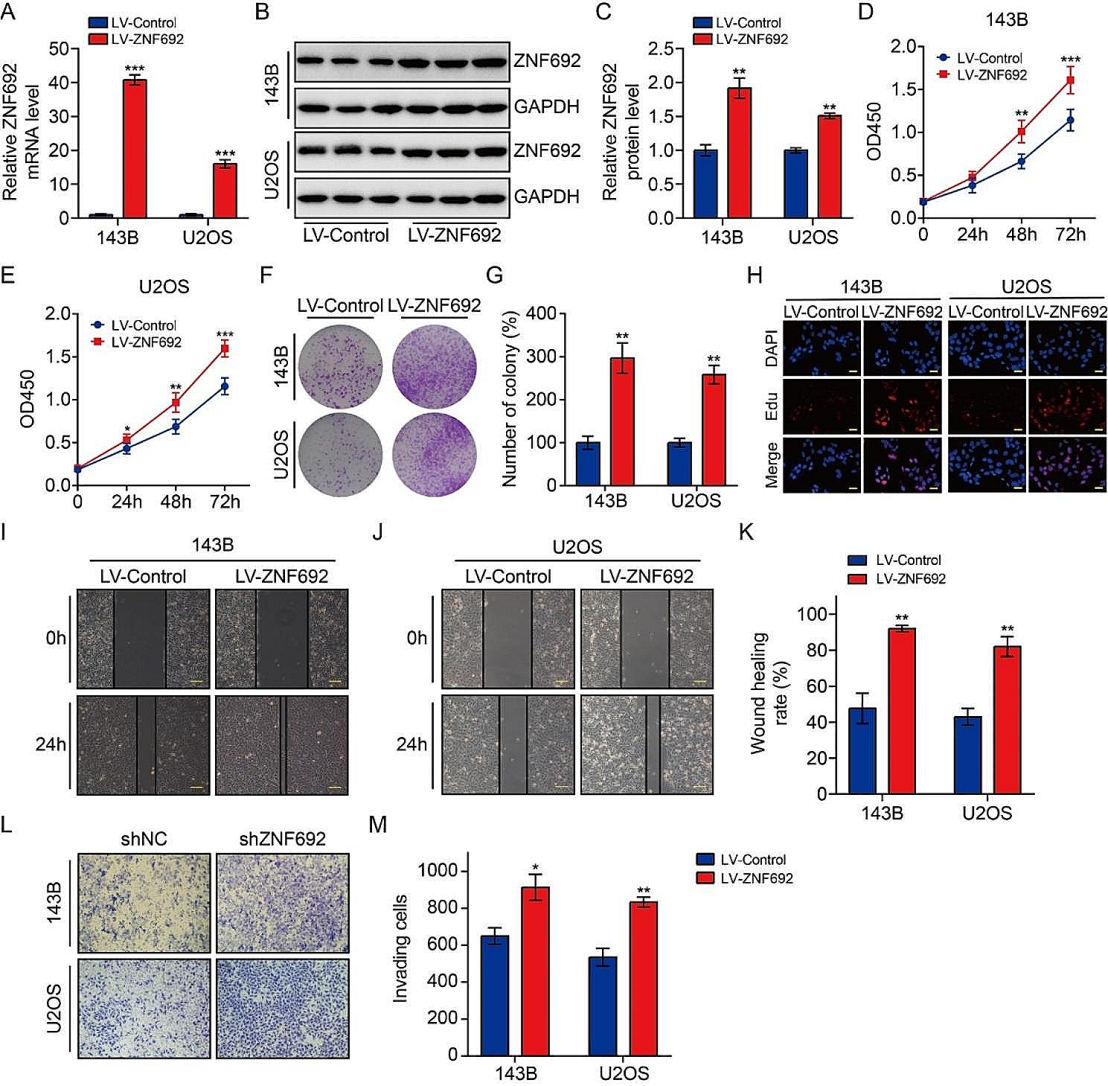
Enforced expression of ZNF692 promotes cell proliferation, migration, and invasion. (**A-C**) Ectopic expression of ZNF692 was determined by qRT-PCR and western blot assays. (**D-E**) CCK-8 assay was performed to compare cell proliferation ability in cells stably overexpressing ZNF692 and control cells. (**F-G**) Representative images and quantitative analysis of colony formation in cells stably overexpressing ZNF692 and control cells. (**H**) The proportion of Edu positive cell was significantly increased in osteosarcoma cells stably overexpressing ZNF692. Scale bar, 200 μm. (**I-K**) Representative images and quantitative analysis of cell migration based on wound healing assay. Scale bar, 200 μm. (**L-M**) Enforced expression of ZNF692 increased cell invasion ability based on transwell assay. Student’s *t*-test and one-way ANOVA were performed to analyze differences between groups. All data are presented as means ± standard deviations (SD). *P < 0.05, **P < 0.01, ***P < 0.001

In order to investigate the impact of ZNF692 on the malignant behavior of osteosarcoma cells, we assessed the influence of ZNF692 overexpression on the proliferation, migration, and invasion of these cells. The transfection effectiveness of the lentivirus overexpressing ZNF692 was confirmed by the use of qRT-PCR and Western blotting techniques, as shown in Fig. 3A-C. The results from CCK-8, colony formation, and Edu incorporation tests indicated that the overexpression of ZNF692 greatly enhanced cell proliferation in 143B and U2OS cells (Fig. 3D-H). The results from wound-healing and transwell invasion studies demonstrated enhanced cell migration and invasion in osteosarcoma cells that had a stable overexpression of ZNF692 (Fig. 3I-M). Overall, our findings suggest that ZNF692 enhances the proliferation, migration, and invasion of osteosarcoma cells in *vitro*.

### ZNF692 promotes osteosarcoma cell proliferation, migration, and invasion through activation of MEK/ERK pathway

**Fig. 4:**
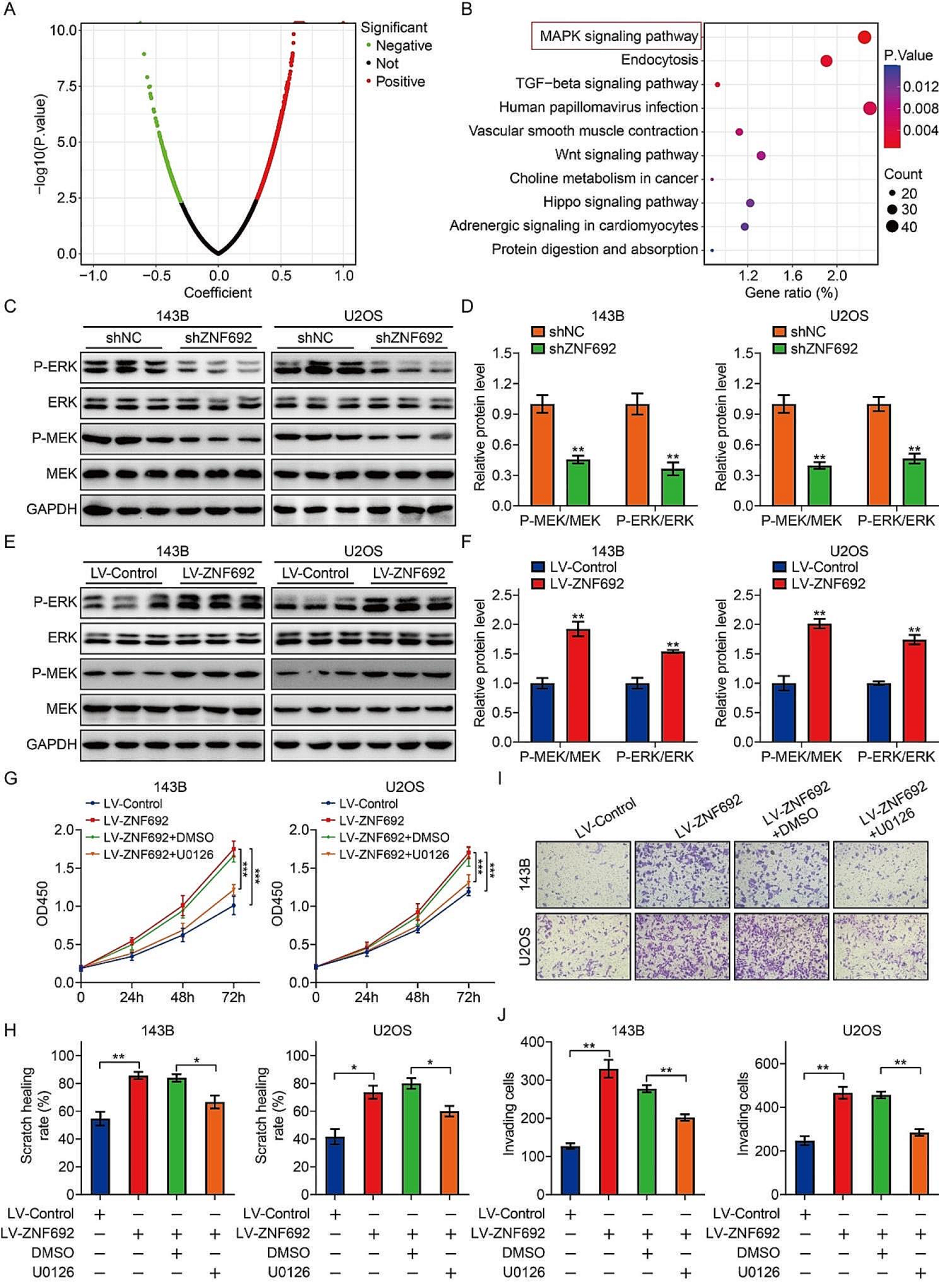
ZNF692 positively regulates MEK/ERK signaling to promote cell proliferation, migration, and invasion. (**A**) Volcano plot illustrating the ZNF692-associated genes based on Pearson correlation analysis. (**B**) KEGG analysis revealed that ZNF692-associated genes were mainly enriched in MAPK signaling pathway. (**C-D**) Western blot analysis of MEK1/2, P-MEK1/2, ERK1/2, and P-ERK1/2 in osteosarcoma cells stably knocking down *ZNF692*, and quantitative analysis. (**E-F**) Western blot analysis of MEK1/2, P-MEK1/2, ERK1/2, and P-ERK1/2 in osteosarcoma cells stably overexpressing ZNF692, and quantitative analysis. (**G**) Application of U0126 (an inhibitor of MEK/ERK signaling) partially counteracted the enhanced cell proliferation generated by overexpression of ZNF692. (**H**) Application of U0126 partially counteracted the enhanced cell migration generated by overexpression of ZNF692. (**I-J**) Application of U0126 partially counteracted the enhanced cell invasion generated by overexpression of ZNF692. Student’s *t*-test and one-way ANOVA were performed to analyze differences between groups. All data are presented as means ± standard deviations (SD). *P < 0.05, **P < 0.01, ***P < 0.001

In order to better understand the molecular mechanism behind the ZNF692-mediated increase in osteosarcoma cell proliferation, migration, and invasion, we conducted Pearson’s correlation analysis on the TCGA osteosarcoma expression matrix to identify the genes related with ZNF692. A total of 2087 genes had a significant association with ZNF692, as shown by a *P*-value of less than 0.05 and an absolute value of correlation coefficient more than 0.3 (Fig. 4A). A KEGG enrichment analysis was performed to identify the associated signaling pathways linked to ZNF692. The findings indicated that the genes associated with ZNF692 were mostly concentrated in the mitogen-activated protein kinase (MAPK) signaling pathway (Fig. 4B). The MAPK pathway is a strongly preserved pathway that comprises three well-known signaling pathways: ERK, p38, and JNK signaling. The MAPK/ERK signaling pathway plays a crucial role in cell proliferation, migration, and invasion. Thus, we investigated the impact of ZNF692 on ERK activation in osteosarcoma cells by analyzing the expression of MEK1/2, P-MEK1/2, ERK1/2, and P-ERK1/2 using Western blotting. Knocking down ZNF692 resulted in a reduction in the protein expression of P-MEK1/2 and P-ERK1/2, as seen in Fig. 4C-D. In contrast, the overexpression of ZNF692 resulted in an elevated production of protein phosphorylation of MEK1/2 and ERK1/2, as seen in Fig. 4E-F. The findings indicate that ZNF692 has a favorable regulatory effect on the MEK/ERK signaling pathway in osteosarcoma cells. In order to provide further evidence for the role of ZNF692-mediated MEK/ERK signaling in promoting osteosarcoma cell proliferation, migration, and invasion, we used U0126, a highly specific inhibitor of MEK/ERK signaling. Our findings indicate that the application of U0126 partially counteracted the enhanced cell proliferation, migration, and invasion generated by overexpression of ZNF692 (Fig. 4G-J).

### TNK2 is a direct transcription target of ZNF692

**Fig. 5:**
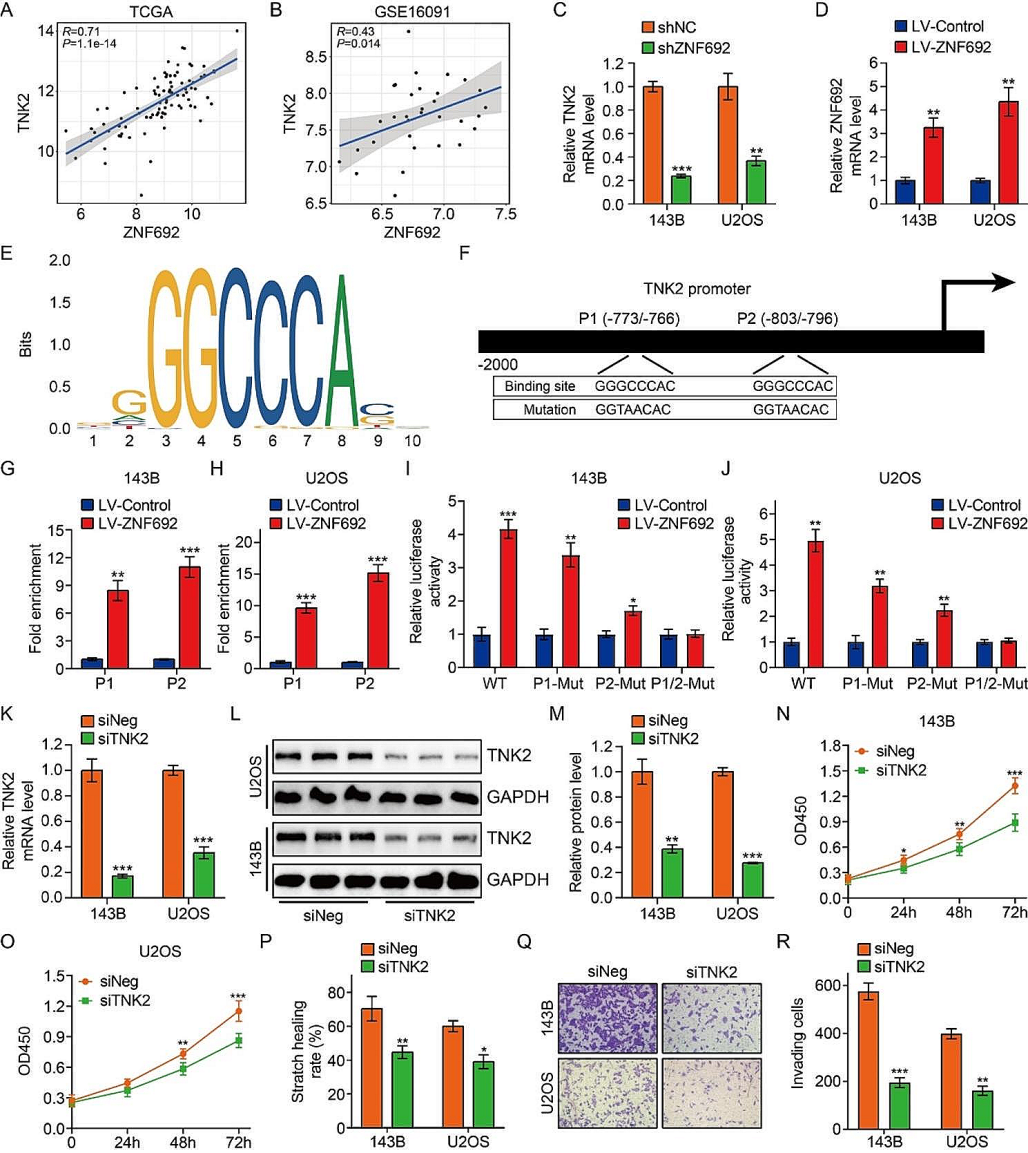
Identification of TNK2 as a direct transcriptional target of ZNF692. (**A-B**) *ZNF692* expression was significantly positively correlated with the expression of *TNK2* based on TCGA and GSE16091 datasets. (**C-D**) qRT-PCR analysis of TNK2 mRNA levels in 143B and U2OS cells stably knocking down or overexpressing ZNF692. (**E**) The binding motif of ZNF692 predicted by JASPAR online tool. (**F**) The two putative ZNF692-binding sites (P1 and P2) in the − 2000 bp to 0 bp sequence upstream of the transcription start site of *TNK2*. (**G-H**) ChIP-qPCR assays in 143B and U2OS cells stably overexpressing ZNF692 confirmed significant enrichment of ZNF692 in the two putative binding sites of *TNK2*. (**I-J**) A luciferase reporter assay suggested that ZNF692 apparently forced *TNK2* promoter activity. Site-specific mutagenesis was used to determine ZNF692-responsive regions in the *TNK2* promoter. (**K-M**) qRT-PCR and western blot assays were performed to assess the knockdown efficiency of siRNA specifically targeting *TNK2*. (**N-R**) CCK-8, wound healing, and transwell invasion assays were conducted to evaluate the effect of TNK2 knock-down on cell proliferation, migration, and invasion. Student’s *t*-test and one-way ANOVA were performed to analyze differences between groups. All data are presented as means ± standard deviations (SD). *P < 0.05, **P < 0.01, ***P < 0.001

Given that ZNF692 was presumed to function as a transcription factor, we conducted a search for downstream targets among the genes linked with *ZNF692* in the Pearson’s analysis. *TNK2* caught our interest due to its involvement in tumor growth and its ability to activate MAPK/ERK signaling. Figure 5A-B demonstrate a strong positive correlation between *ZNF692* expression and *TNK2* expression, as shown in the gene expression matrices of the TCGA and GSE16091 osteosarcoma cohorts. The GEPIA database (http://gepia.cancer-pku.cn/detail.php) also yielded comparable findings across several tumor types, including ACC, BLCA, BRCA, and others (Supplementary Fig. 1A-T). The qRT-PCR findings demonstrated that the downregulation of ZNF692 in 143B and U2OS cells led to a decrease in the expression of TNK2, as seen in Fig. 5C. Conversely, the excessive production of ZNF692 in osteosarcoma cells resulted in an elevated expression of TNK2, as seen in Fig. 5D. In summary, our findings indicate that ZNF692 may have a favorable regulatory effect on TNK2. In order to ascertain the direct targeting of TNK2 by ZNF692, we examined the promoter sequence of *TNK2* and discovered two potential binding sites (P1 and P2) for ZNF692. These sites were located in the region spanning from − 2000 bp to 0 bp upstream of the transcription start site of *TNK2*, using the JASPAR online tool for this analysis (Fig. 5E-F). Direct binding of the *TNK2* promoter by ZNF692 was confirmed by anti-ZNF692 ChIP assay and it showed that enriched binding within regions of the two putative binding sits predicted by JASPAR (Fig. 5G-H). Additionally, the luciferase reporter experiment demonstrated that ZNF692 enhanced the activity of the *TNK2* promoter. Through site-specific mutagenesis, it was shown that mutating one of the two presumed binding sites resulted in decreased luciferase activity. Furthermore, simultaneous mutation of both binding sites entirely abolished luciferase activity, as shown in Fig. 5I-J. These findings indicate that *TNK2* is a direct transcriptional target of ZNF692. In order to investigate the function of TNK2 in osteosarcoma, we developed a targeted siRNA to inhibit TNK2 and created a plasmid to overexpress TNK2. We next examined the impact of TNK2 knockdown or overexpression on the malignant properties of osteosarcoma cells. The transfection efficiencies were validated using qRT-PCR and Western blot analysis (Fig. 5K-M and Supplementary Fig. 2A-C). Figure 5N-R demonstrate that the inhibition of TNK2 had a considerable negative impact on cell proliferation, migration, and invasion. In contrast, the overexpression of TNK2 resulted in an increased rate of cell proliferation, migration, and invasion, as seen in Supplementary Fig. 2D-H. In addition, the Kaplan-Meier survival analysis conducted on the TCGA osteosarcoma cohort indicated that elevated TNK2 expression was linked to a negative prognosis in patients with osteosarcoma (Supplementary Fig. 2I).

### ZNF692 activates the ERK/MEK pathway by increasing TNK2 expression

**Fig. 6:**
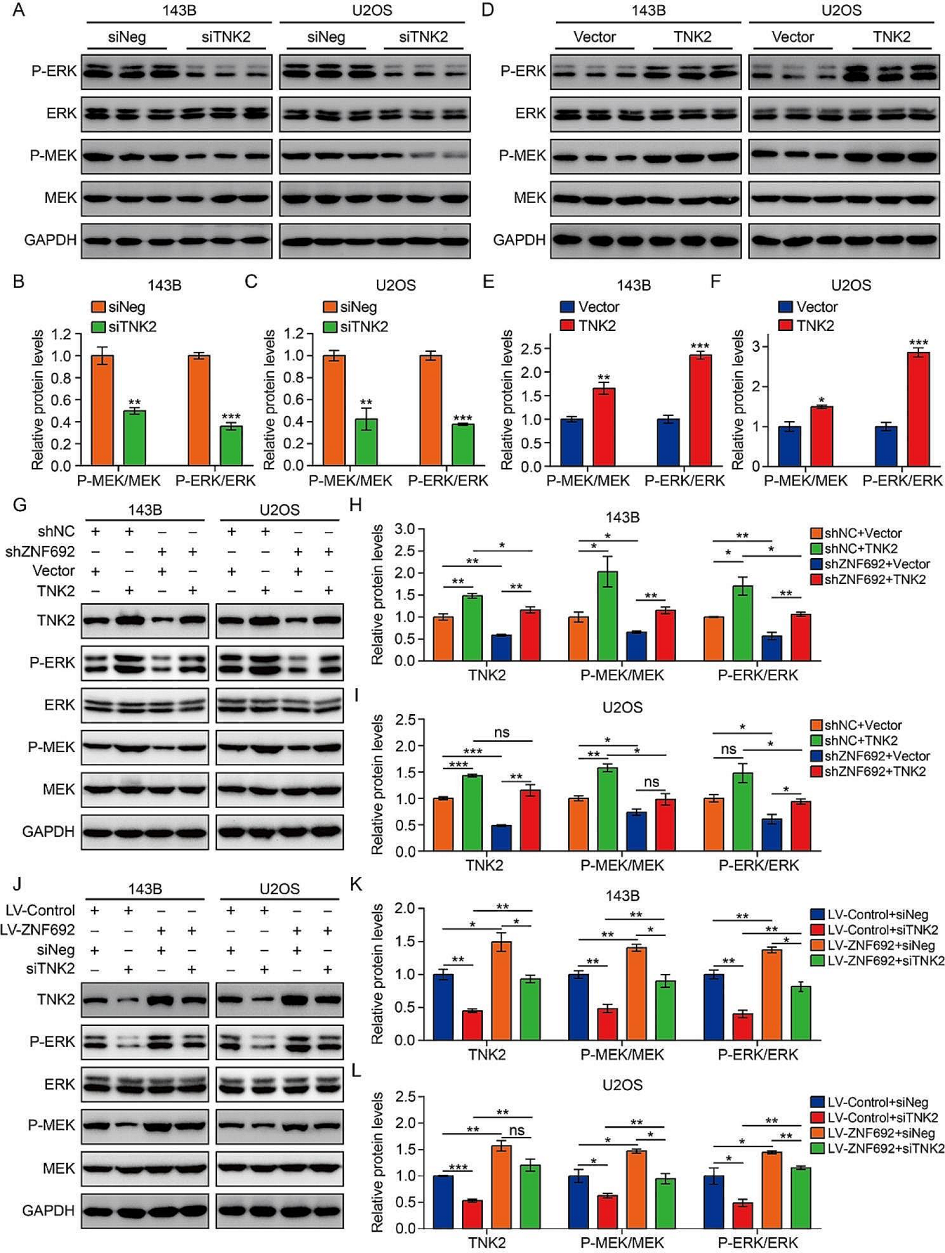
TNK2 is essential for ZNF692-mediated activation of MEK/ERK signaling pathway. (**A-C**) Western blot and quantitative analyses of MEK1/2, P-MEK1/2, ERK1/2, and P-ERK1/2 in osteosarcoma cells transfected with siRNA specifically targeting TNK2 (siTNK2) and negative control (siNeg). (**D-F**) Western blot and quantitative analyses of MEK1/2, P-MEK1/2, ERK1/2, and P-ERK1/2 in osteosarcoma cells transfected with empty vector and TNK2 overexpression plasmid. (**G-I**) Western blot and quantitative analyses of MEK1/2, P-MEK1/2, ERK1/2, and P-ERK1/2 for the indicated groups revealed that enforced expression of TNK2 attenuated inhibitory effect of ZNF692 knockdown on P-MEK1/2 and P-ERK1/2 (- and + stand for categorical values absent and present, referring to gene knockdown or overexpression by means of the listed constructs) (**J-L**) Western blot and quantitative analyses of MEK1/2, P-MEK1/2, ERK1/2, and P-ERK1/2 for the indicated groups revealed that silencing TNK2 in osteosarcoma cells partially reversed promoting effect of ZNF692 overexpression on P-MEK1/2, P-ERK1/2. Student’s *t*-test and one-way ANOVA were performed to analyze differences between groups. All data are presented as means ± standard deviations (SD). *P < 0.05, **P < 0.01, ***P < 0.001

Prior research indicated that TNK2 had a favorable regulatory effect on the MEK/ERK signaling pathway [27]. In order to validate this in osteosarcoma cells, we measured the protein levels of MEK1/2, P-MEK1/2, ERK1/2, and P-ERK1/2 in 143B and U2OS cells that were either depleted or overexpressed with TNK2. Figure 6A-C demonstrate that the knock-down of TNK2 led to a decrease in the phosphorylation of MEK1/2 and ERK1/2. In contrast, the overexpression of TNK2 resulted in an increased levels of P-MEK1/2 and P-ERK1/2, as seen in Fig. 6D-F. In order to investigate the involvement of TNK2 in the activation of the MEK/ERK signaling pathway by ZNF692, we altered the expression of TNK2 in osteosarcoma cells with either reduced or increased levels of ZNF692. The Western blot analysis indicated that the enforced expression of TNK2 reduced the inhibitory impact of ZNF692 knockdown on the levels of P-MEK1/2 and P-ERK1/2 (Fig. 6G-I). In the same way, the inhibition of TNK2 in osteosarcoma cells partly counteracted the enhancing impact of ZNF692 overexpression on P-MEK1/2 and P-ERK1/2 (Fig. 6J-L). The functional assays demonstrated that the suppressive impact of ZNF692 knockdown on cell proliferation, migration, and invasion was notably reduced when TNK2 overexpression was introduced (Supplementary Fig. 3A-B, 3E, 3G-H). In contrast, the inhibition of TNK2 hindered the ability of ZNF692 overexpression to promote cell proliferation, migration, and invasion (Supplementary Fig. 3C-D, 3 F, 3I-J).

### Reduced expression of ZNF692 inhibits tumor growth in vivo

**Fig. 7:**
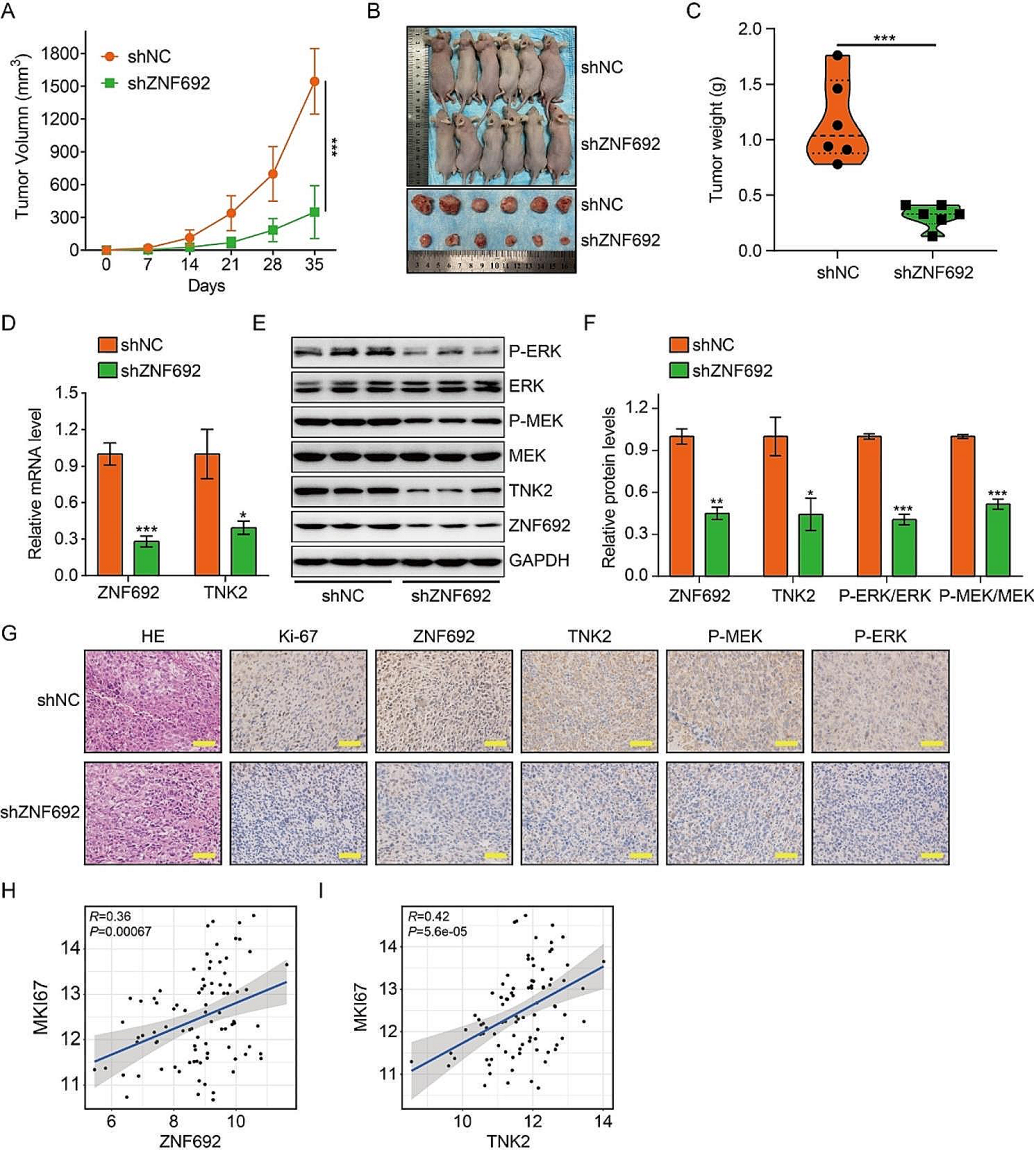
Knocking down of ZNF692 inhibits tumor growth in vivo. (**A**) Tumor volume was monitored every week to compare tumor growth in vivo. (**B**) Images of xenograft tumors formed in nude mice subcutaneously injected with 143B cells stably knocking down ZNF692 and control cells. (**C**) Tumor weight was compared between the two groups. (**D**) qRT-PCR analyses of ZNF692 and TNK2 mRNA levels in xenograft tumors. (**E-F**) Western blot and quantitative analyses of ZNF692, TNK2, MEK1/2, P-MEK1/2, ERK1/2, and P-ERK1/2 in xenograft tumors. (**G**) IHC analysis of the expression of Ki-67, ZNF692, TNK2, P-MEK1/2, and P-ERK1/2 in xenograft tumors induced by cells stably knocking down ZNF692 or control cells. Scale bar, 200 μm. (**H-I**) The expression of *ZNF692* and *TNK2* were positively correlated with the expression *Mki67* based on TCGA dataset. Scale bar: 200 µM. Student’s *t*-test and one-way ANOVA were performed to analyze differences between groups. All data are presented as means ± standard deviations (SD). *P < 0.05, **P < 0.01, ***P < 0.001

In order to examine the impact of ZNF692 on the proliferation of osteosarcoma cells in *vivo*, we subcutaneously injected 143B cells that had been genetically modified to suppress ZNF692, as well as control cells, into nude mice. Figure 7A-C demonstrate that tumors created by 143B cells with stable expression of shZNF692 had a decreased average tumor volume and weight compared to tumors induced by control cells. This indicates that the suppression of ZNF692 led to a decrease in tumor development in *vivo*. Subsequently, in order to further validate the impact of ZNF692 on MEK/ERK signaling via transcriptional regulation of TNK2, we assessed the levels of ZNF692 and TNK2 expression, as well as the degree of phosphorylation of MEK1/2 and ERK1/2 in the xenograft tumors. The qRT-PCR findings indicated that the mRNA levels of ZNF692 and TNK2 were reduced in the tumor formed by cells with suppressed *ZNF692* compared to the tumors induced by control cells (Fig. 7D). The levels of ZNF692, TNK2, p-MEK1/2, and p-ERK1/2 were found to be considerably lower in ZNF692-knockdown tumors compared to control tumors, as shown by Western blot and IHC analysis (Fig. 7E-G). Furthermore, the immunohistochemistry (IHC) findings demonstrated a reduced proportion of Ki-67-positive cells in the tumor that had undergone stable knockdown of ZNF692, compared to the control tumors (Fig. 7G). In addition, Pearson’s correlation analysis demonstrated a significant association between the expression of *ZNF692 and TNK2* with the expression of *Mki67* (Fig. 7H-I). Collectively, our findings indicate that the decreased expression of ZNF692 hampers the development of tumors via deactivating TNK2-mediated MEK/ERK signaling.

### ZNF692-TNK2 axis could be regarded as prognostic biomarker in osteosarcoma

**Fig. 8:**
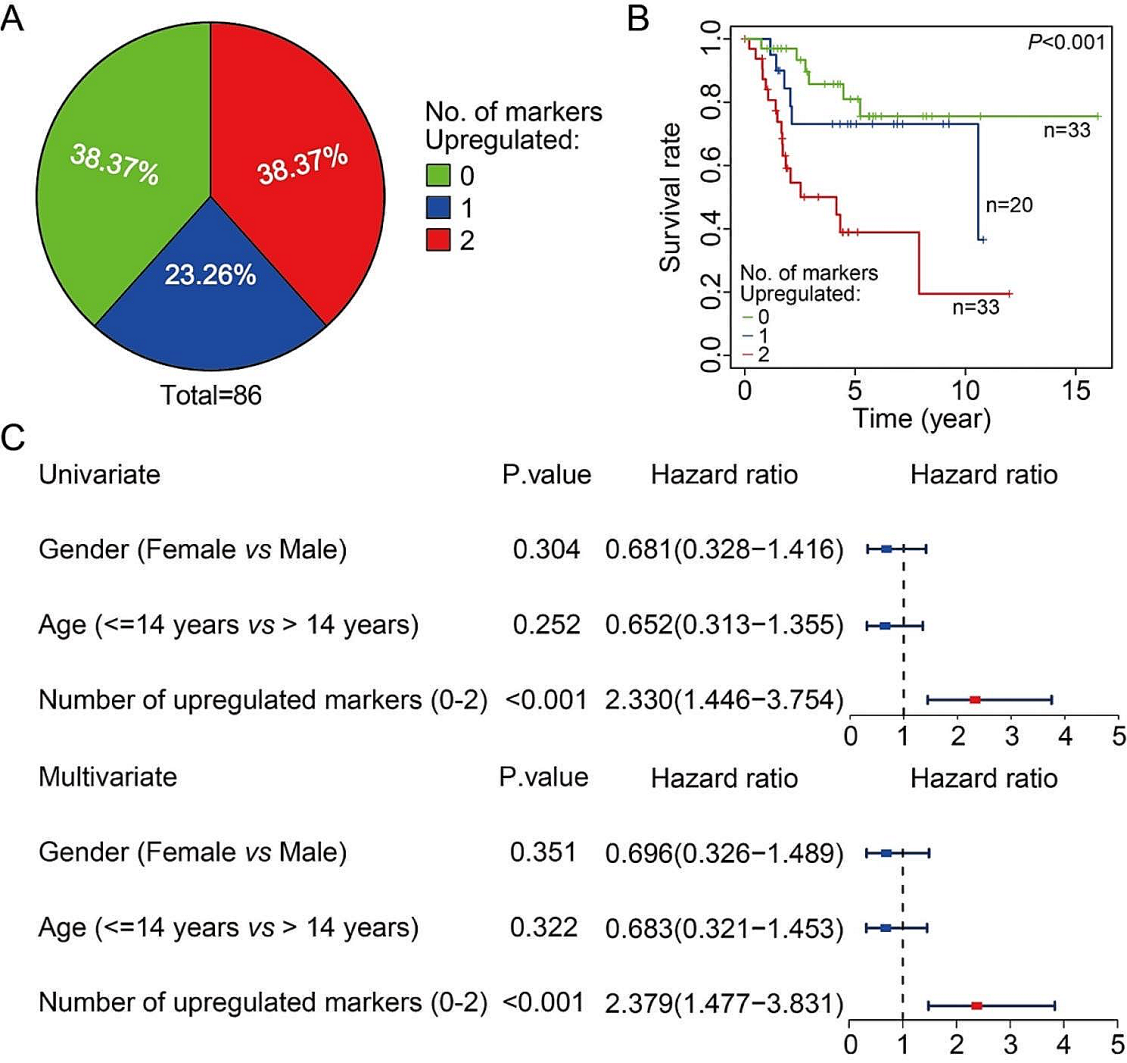
ZNF692-TNK2 axis is an attractive candidate as a prognostic biomarker of osteosarcoma. (**A**) The proportion of patients in different sub-groups stratified by the number of up-regulated marker genes. *ZNF692* and *TNK2* expression was stratified by the corresponding medians by RNA-sequencing data from TCGA dataset, and the patients were divided into three groups as indicated. (**B**) Kaplan-Meier analysis of overall survival for osteosarcoma patients based on the number of upregulated marker genes. (**C**) Univariate and multivariate Cox regression analysis of the number of up-regulated makers and clinical features including gender and age in patients with osteosarcoma in TCGA database

Ultimately, we investigated the clinical significance of the ZNF692-TNK2 axis in patients with osteosarcoma using data from the TCGA database. We created a composite panel by integrating *ZNF692* and *TNK2* to forecast the prognosis of osteosarcoma. Patients were categorized into several subgroups according to the quantity of up-regulated marker genes, and the distribution of patients in each subgroup is shown in Fig. 8A. The Kaplan-Meier survival analysis and the log-rank test indicated that patients who had increased expression of both markers had the shortest overall survival. However, there was no statistically significant difference in prognosis between the group with up-regulated expression of only one marker and the group with low expression of both *ZNF692* and *TNK2* (Fig. 8B). Furthermore, both univariate and multivariate Cox regression analyses demonstrated that the quantity of elevated markers might function as an independent prognostic factor for assessing the survival of osteosarcoma patients (Fig. 8C). Hence, the ZNF692-TNK2 axis may be potential prognostic biomarker for osteosarcoma.

## Discussion

Zinc finger proteins have been extensively researched for their crucial functions as transcriptional regulators in physiological processes such as cell proliferation, differentiation, development, and cell death [28–30]. Recent research has shown a growing correlation between the disruption of zinc finger proteins and the development and progression of tumors [31–33]. ZNF692, a member of the Krüppel C2H2‑type zinc finger protein family, has five C2H2‑type zinc finger repeats, which enable ZNF692 to attach to the promoter regions of certain genes and regulate their transcription [16]. Despite the results revealed ZNF692 exhibited tumor-promoting activity in malignancies like LUAD, COAD, and cervical cancer, its significance and precise mechanism in osteosarcoma still has not been clarified untill now. Our work demonstrated that *ZNF692* functions as an oncogene in osteosarcoma. Initially, we discovered that ZNF692 exhibited an increase in expression in osteosarcoma tissues and cell lines. Besides, a strong correlation was seen between elevated levels of ZNF692 and worse prognosis in individuals diagnosed with osteosarcoma. Therefore, *ZNF692* might function as a risk factor and an independent prognostic biomarker in osteosarcoma. Furthermore, the absence of ZNF692 resulted in a reduction of the proliferation, migration, and invasion of osteosarcoma cells, and hindered tumor growth in *vivo*. In contrast, the overexpression of ZNF692 stimulated proliferation, migration, and invasion, providing more evidence for its role in driving tumor development. Thus, our research reveals that *ZNF692* is a potentially new prognostic predictor and an oncogenic factor osteosarcoma.

In order to gain a deeper understanding of how ZNF692 facilitates cell proliferation, migration, and invasion, and taking into account the possibility that ZNF692 acts as a transcriptional regulator to control its downstream targets, we performed Pearson’s correlation analysis to identify genes that exhibited a significant correlation with *ZNF692*. Subsequently, KEGG enrichment analysis indicated a high enrichment of MAPK signaling. This finding provides evidence for the regulatory function of ZNF692 in the MAPK pathway. The canonical MAPK/ERK cascade is the primary signaling route across all MAPK pathways and garners significant interest owing to its dual function in controlling tumor cell survival, dissemination, and resistance to medication treatment [34–36]. The MAPK/ERK pathway has been shown to serve as a tumor suppressor and the more common pro-oncogenic signal [37–39]. In order to clarify the function of ZNF692 in MAPK/ERK signaling, we measured the levels of phosphorylation of MEK1/2 and EKR1/2. Our findings demonstrate that ZNF692 has a favorable effect in activating the MEK/ERK cascade. In addition, we discovered that U0126, a small molecule inhibitor of MEK/ERK signaling, partially counteracted the impacts of ZNF692 overexpression on the proliferation, migration, and invasion of osteosarcoma cells. Thus, it may be inferred that ZNF692 enhances the proliferation, migration, and invasion of osteosarcoma cells by stimulating the MEK/ERK signaling pathway.

*TNK2*, situated on chromosome 3q29, encodes a remarkably conserved non-receptor tyrosine kinase, also referred to as the activated Cdc42-associated tyrosine kinase 1 (*ACK1*) [40]. The deregulated TNK2 acts as a carcinogenic factor and is associated with tumor progression and patients’ overall survival [41–44]. Disruption of TNK2 leads to reduced cell growth, halted cell cycle progression, increased apoptosis, and heightened susceptibility to radiation [45–48], suggesting that TNK2 has promise as a potential target for treating malignant tumors. Here, we have provided data that supports the oncogenic role of TNK2 in osteosarcoma. In addition, an increased expression of *TNK2* was shown to correlate with a worse overall survival rate in patients, indicating that *TNK2* might serve as a promising prognostic biomarker for osteosarcoma. Further investigation is required to assess the prognostic prediction ability of *TNK2* in a larger external validation cohort. Given the observed strong positive correlation between *TNK2* expression and the expression of *ZNF692* in different tumor tissues, as well as the regulation of *TNK2* following ZNF692 knock-down or overexpression in osteosarcoma cells, we hypothesized that *TNK2* may be a direct transcriptional target of ZNF692. The use of luciferase reporter assay and ChIP assay provides confirmation that ZNF692 increases the transcription of *TNK2* in osteosarcoma cells through binding to the promotor region.

Previous studies reported that the absence of TNK2 in lung cancer cells had an impact on MAPK signaling based on RNA-Seq analysis [49]. In primary neurons and PC12 cells, TNK2 was shown to function as an upstream regulator of MAPK/ERK signaling and enhance the phosphorylation of ERK1/2 [50]. Pharmacological suppression of TNK2 prevented the activation of MAPK/ERK signaling in juvenile myelomonocytic leukemia (JMML) and reduced the size of the tumor [51]. Here, our findings indicate that the inhibition of TNK2 led to a decrease in the phosphorylation of MEK1/2 and ERK1/2. In contrast, the excessive expression of TNK2 resulted in an elevation in P-MEK1/2 and P-ERK1/2 levels. Collectively, our findings, along with the aforementioned studies, consistently demonstrate that TNK2 has a positive regulatory influence on the MAPK/ERK signaling pathway. To further investigate the role of TNK2 on ZNF692-mediated activation of ERK signaling and increased malignant behaviors, we manipulated the expression of TNK2 in cells that either had ZNF692 knocked down or overexpressed. Our findings revealed that the enforced expression of TNK2 weakened the inhibitory effect of ZNF692 knockdown on P-MEK1/2 and P-ERK1/2. Conversely, knocking down TNK2 partially reversed the promoting effect of ZNF692 overexpression on P-MEK1/2 and P-ERK1/2. The functional studies demonstrated that the suppressive impact of ZNF692 knockdown on cell proliferation, migration, and invasion was notably weakened when TNK2 was overexpressed. In contrast, the reduction of TNK2 hindered the ability of ZNF692 overexpression to enhance cell proliferation, migration, and invasion. Collectively, our findings indicate that ZNF692 enhances the proliferation, migration, and invasion of osteosarcoma cells by activating the MEK/ERK pathway via TNK2. Nevertheless, the precise method via which TNK2 facilitates the activation of ERK signaling remains unclear. Several downstream proteins of TNK2, including AKT, STAT3, WWOX, and WASP, have been extensively described [45, 52–54]. TNK2 could bind to these proteins and phosphorylate them at special sites. Thus, TNK2 might phosphorylate the upstream kinase in this pathway, so initiating the activation of MAPK/ERK signaling. Alternatively, the activation of MAPK/ERK signaling may have been caused by the intercellular signaling cross-talk resulting from the overexpression of TNK2. For instance, upregulation of TNK2 may strengthen the stability of the EGF receptor, hence activating the MAPK/ERK cascade in response to EGF stimulation [55, 56]. Additional experiments should be devised to facilitate deeper investigation.

In conclusion, our investigation elucidated the pro-tumorigenic function of ZNF692 in osteosarcoma. Increased expression of *ZNF692* is a reliable predictor of worse overall survival in osteosarcoma patients. ZNF692 enhances the proliferation, migration, and invasion of osteosarcoma cells via TNK2-dependent stimulation of the MEK/ERK signaling pathway. ZNF692 might play a role as a potential predictive biomarker and a promising target for novel therapeutics in osteosarcoma in the future.

### Electronic supplementary material

Below is the link to the electronic supplementary material.

Supplementary Material 1

Supplementary Material 2

Supplementary Material 3

## Data Availability

Data related to the current study are available from the corresponding author on reasonable request.

## Abbreviations

TCGA: The Cancer Genome Atlas
GEO: Gene Expression Omnibus
KEGG: Kyoto Encyclopedia of Genes and Genomes
DAVID: Database for Annotation, Visualization, and Integrated Discovery
ROC: Receiver operating characteristic
AUC: Area under the curve
BLCA: Bladder Urothelial Carcinoma
BRCA: Breast invasive carcinoma
CHOL: Cholangiocarcinoma
COAD: Colon adenocarcinoma
ESCA: Esophageal carcinoma
HNCS: Head and Neck squamous cell carcinoma
KIRC: Kidney renal clear cell carcinoma
KIRP: Kidney renal papillary cell carcinoma
LIHC: Liver hepatocellular carcinoma
LUAD: Lung adenocarcinoma
LUSC: Lung squamous cell carcinoma
PRAD: Prostate adenocarcinoma
READ: Rectum adenocarcinoma
STAD: Stomach adenocarcinoma
UCEC: Uterine Corpus Endometrial Carcinoma
