# Supplementary material for: ZNF692 promotes osteosarcoma cell proliferation, migration, and invasion through TNK2-mediated activation of the MEK/ERK pathway
Source: Biol Direct. 2024 Apr 22;19:28. doi: 10.1186/s13062-024-00472-3 (PMC11034355; doi:10.1186/s13062-024-00472-3)
Supplement: Supplementary file 1 — Supplementary Material 1 [file 13062_2024_472_MOESM1_ESM.docx]

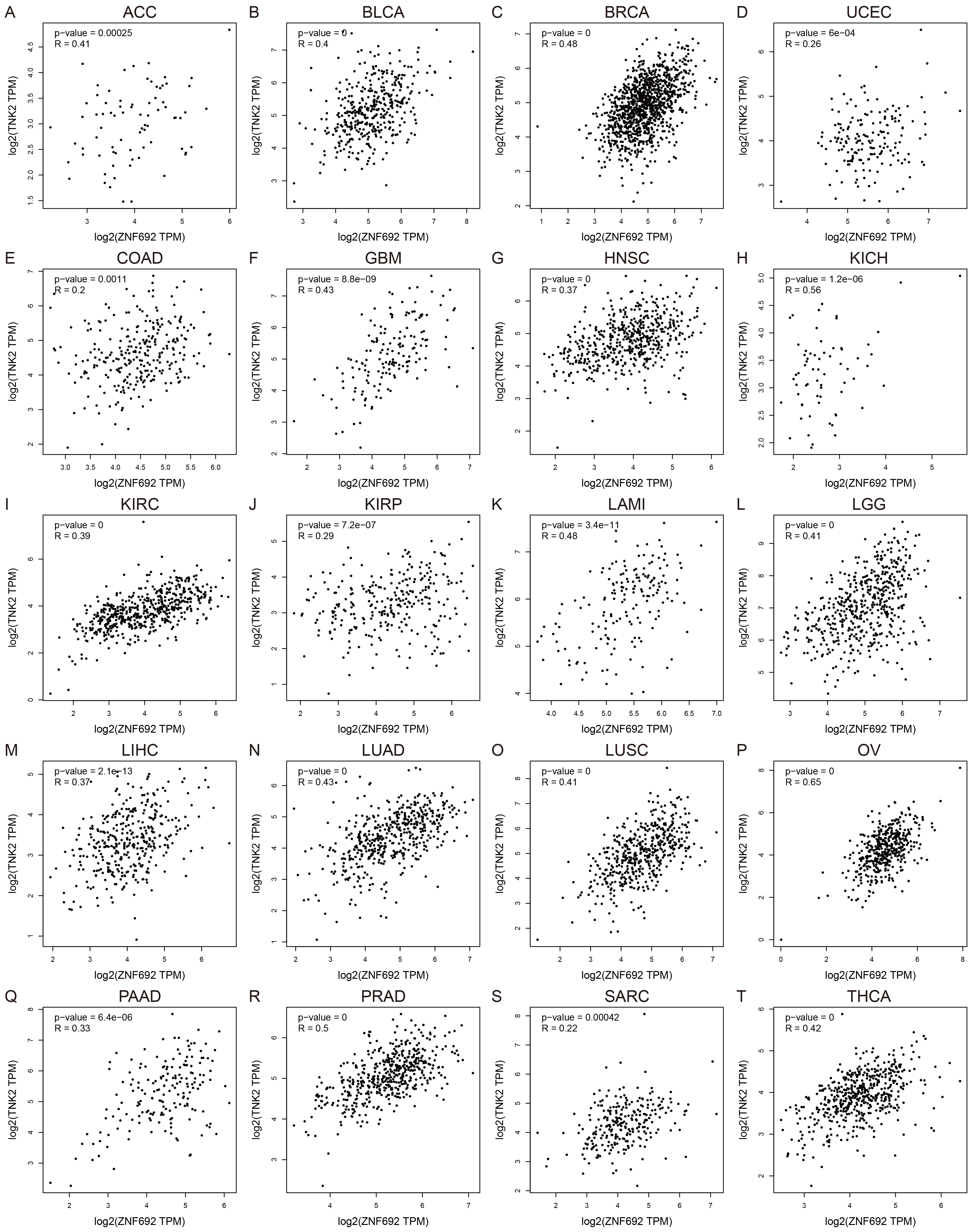


**Supplementary Figure 1.** The expression of ZNF692 was significantly positively associated with TNK2 in tumors including ACC (A), BLCA (B), BRCA (C), UCEC (D), COAD (E), GBM (F), HNSC (G), KICH (H), KIRC (I), KIRP (J), LAMI (K), LGG (L), LIHC (M), LUAD (N), LUSC (O), OV (P), PAAD (Q), PARD (R), SARC (S), and THCA (T) based on GEPIA database.


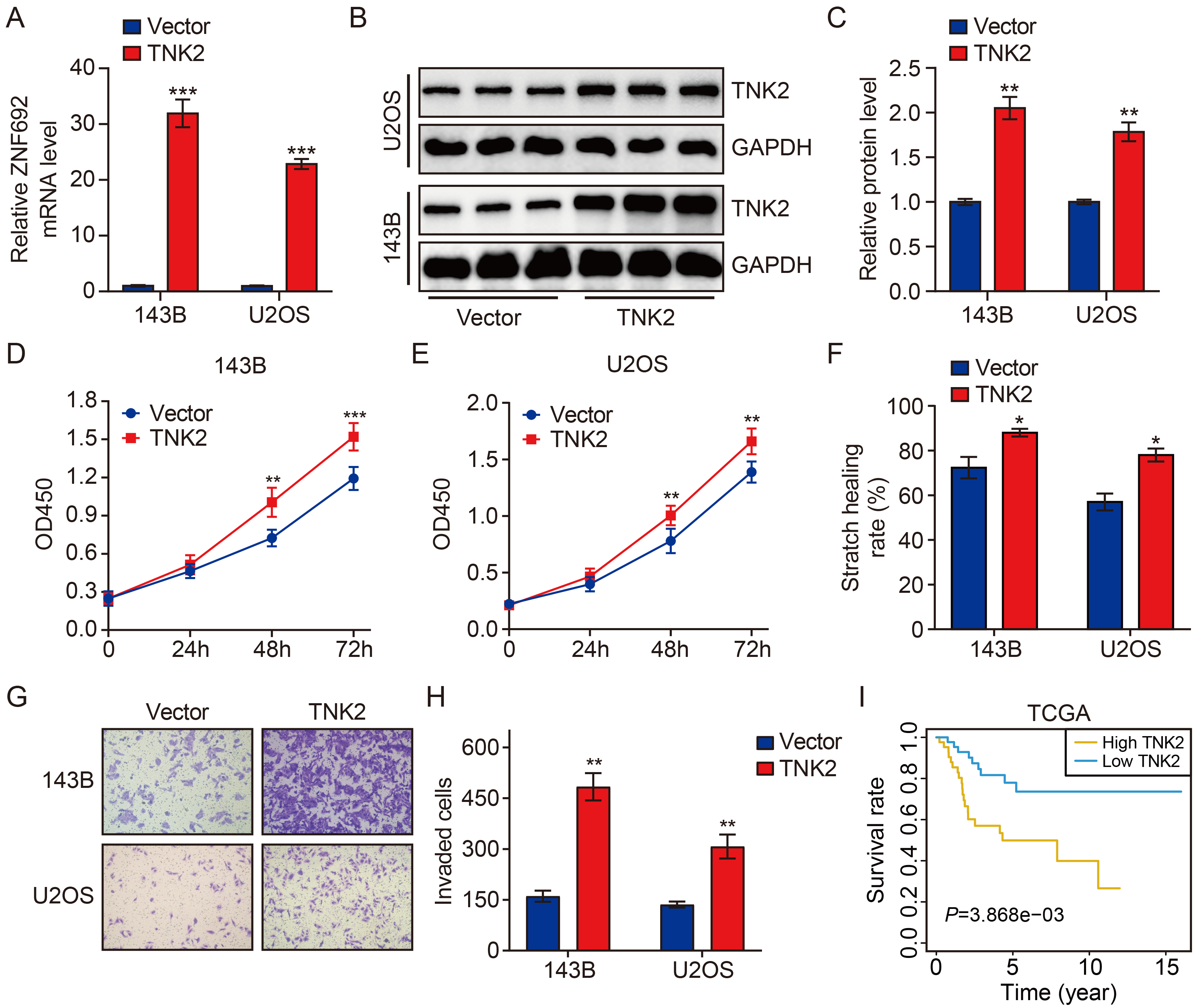


**Supplementary Figure 2.** TNK2 promotes cell proliferation, migration, and invasion in osteosarcoma cells. (A-C) Overexpression of TNK2 was determined by qRT-PCR and western blot assays. (D-H) CCK-8, wound healing, and transwell invasion assays were conducted to evaluate the effect of TNK2 overexpression on cell proliferation, migration, and invasion. (I) Kaplan–Meier survival analysis of TNK2 in osteosarcoma based on TCGA dataset. Student’s t-test and one-way ANOVA were performed to analyze differences between groups. All data are presented as means ± standard deviations (SD). ^*^*P*<0.05, ^**^*P* <0.01, ^***^*P* <0.001.


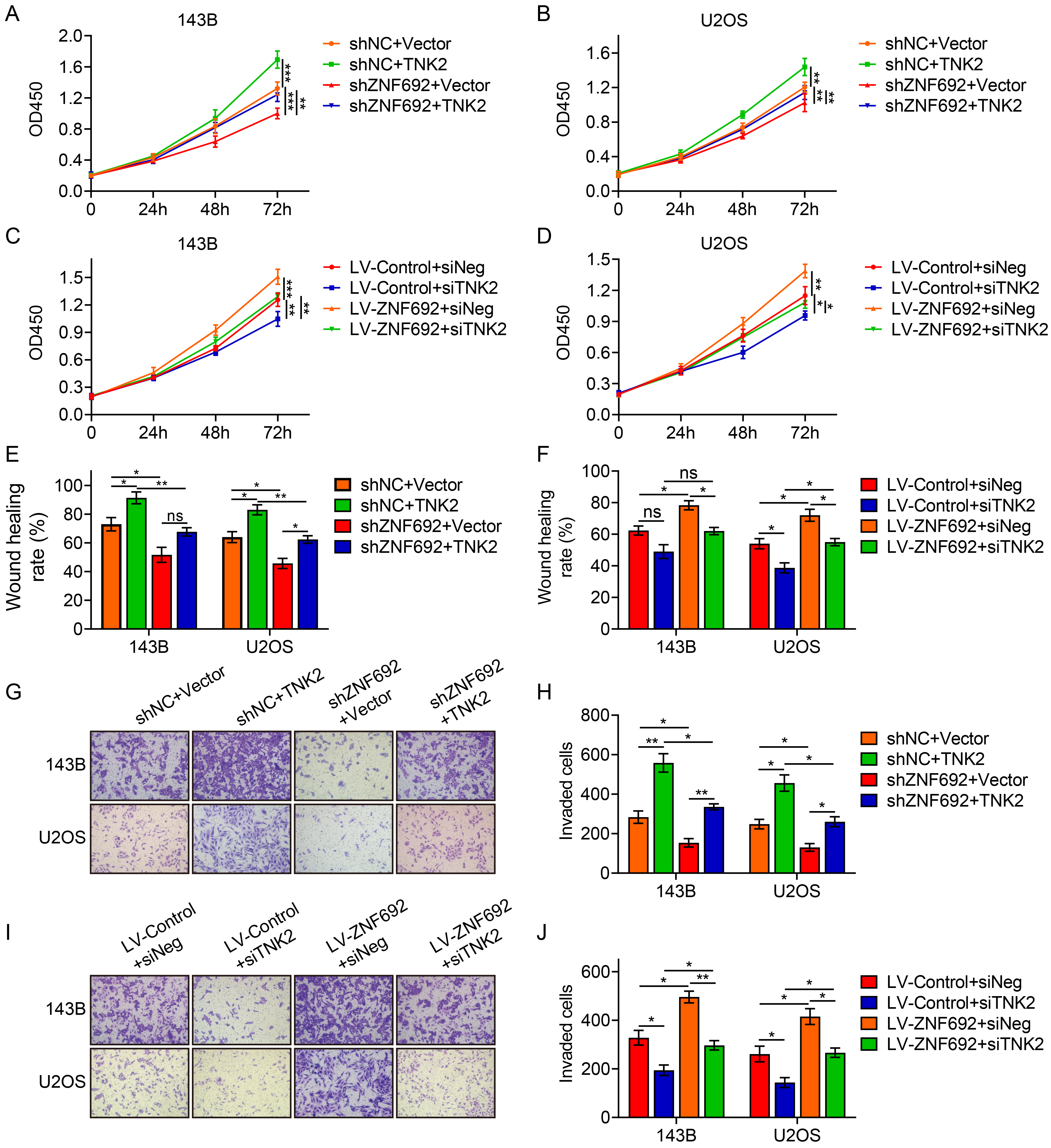


**Supplementary Figure 3.** TNK2 mediated the effect of ZNF692 on cell proliferation, migration, and invasion. (A-B) CCK-8 assay showed that overexpression of TNK2 attenuated the inhibitory effect of ZNF692 knockdown on cell proliferation. (C-D) CCK-8 assay showed that silence of TNK2 partially reversed the promoting effect of ZNF692 overexpression on cell proliferation. (E-F) Wound healing assays were performed after TNK2 overexpression or knock-down in cells stably knocking down or overexpressing ZNF692. (G-J) Transwell assays were performed after TNK2 overexpression or knock-down in cells stably knocking down or overexpressing ZNF692. Student’s t-test and one-way ANOVA were performed to analyze differences between groups. All data are presented as means ± standard deviations (SD). ^*^*P*<0.05, ^**^*P* <0.01, ^***^*P* <0.001.
