# Supplementary material for: ZNF692 promotes osteosarcoma cell proliferation, migration, and invasion through TNK2-mediated activation of the MEK/ERK pathway
Source: Biol Direct. 2024 Apr 22;19:28. doi: 10.1186/s13062-024-00472-3 (PMC11034355; doi:10.1186/s13062-024-00472-3)
Supplement: Supplementary file 2 — Supplementary Material 2 [file 13062_2024_472_MOESM2_ESM.docx]

Supplementary Table 1. Primers used for qRT-PCR

| Primer name | Sequence |
| --- | --- |
| *GAPDH* sense | 5’-CTGAGTACGTCGTGGAGTCC-3’ |
| *GAPDH* antisense | 5’-GTCTTCTGGGTGGCAGTGAT-3’ |
| *ZNF692* sense | 5’-TGATGCCTTGTGACTTCCCT-3’ |
| *ZNF692* antisense | 5’-GCTTCATGTGCTCCTTCAGG-3’ |
| *TNK2* sense | 5’-CGAAACCTCATCCGCCTCTA-3’ |
| *TNK2* antisense | 5’-AGCCAGGTCACGGTGAATAA-3’ |
