# Supplementary material for: ZNF692 promotes osteosarcoma cell proliferation, migration, and invasion through TNK2-mediated activation of the MEK/ERK pathway
Source: Biol Direct. 2024 Apr 22;19:28. doi: 10.1186/s13062-024-00472-3 (PMC11034355; doi:10.1186/s13062-024-00472-3)
Supplement: Supplementary file 3 — Supplementary Material 3 [file 13062_2024_472_MOESM3_ESM.docx]

Supplementary Table 2. Primers used for CHIP-PCR in the TNK2 promoter

| Primer name | Sequence |
| --- | --- |
| binding site 1 sense | 5’-GAGCCCTGCGCTGCCGGA-3’ |
| binding site 1 antisense | 5’-CTCCTGCACCGCCGGGCC-3’ |
| binding site 2 sense | 5’-GAGGAGGGCGGGGTGGGC-3’ |
| binding site 2 antisense | 5’-CCGTCCACCACGGCGGGC-3’ |
